# Comparing CHA_2_DS_2_-VA and CHA_2_DS_2_-VASc scores for stroke risk stratification in patients with atrial fibrillation: a temporal trends analysis from the retrospective Finnish AntiCoagulation in Atrial Fibrillation (FinACAF) cohort

**DOI:** 10.1016/j.lanepe.2024.100967

**Published:** 2024-06-10

**Authors:** Konsta Teppo, Gregory Yoke Hong Lip, Kari Eino Juhani Airaksinen, Olli Halminen, Jari Haukka, Jukka Putaala, Pirjo Mustonen, Miika Linna, Juha Hartikainen, Mika Lehto

**Affiliations:** a Heart Center, Turku University Hospital and University of Turku, Turku, Finland; b Liverpool Centre for Cardiovascular Science at University of Liverpool, Liverpool John Moores University and Liverpool Heart & Chest Hospital, Liverpool, United Kingdom; c Department of Industrial Engineering and Management, Aalto University, Espoo, Finland; d Faculty of Medicine, University of Helsinki, Finland; e Neurology Department, Helsinki University Hospital and University of Helsinki, Helsinki, Finland; f University of Eastern Finland, Kuopio, Finland; g Heart Center, Kuopio University Hospital and University of Eastern Finland, Finland; h Jorvi Hospital, Department of Internal Medicine, Finland; i HUS Helsinki University Hospital and University of Helsinki, Helsinki, Finland; j Danish Center for Health Services Research, Department of Clinical Medicine, Aalborg University, Aalborg, Denmark; k Aalto University, Espoo, Finland

**Keywords:** Atrial fibrillation, CHA_2_DS_2_-VASc, CHA_2_DS_2_-VA, Stroke, Risk prediction

## Abstract

**Background:**

Contemporary data have shown a decrease in the ischaemic stroke risk associated with female sex in patients with atrial fibrillation (AF). We evaluated temporal trends in the predictive value of a non-sex CHA_2_DS_2_-VASc risk score (ie. CHA_2_DS_2_-VA).

**Methods:**

The FinACAF study covers all patients with incident AF between 2007 and 2018 in Finland from all levels of care. The CHA_2_DS_2_-VA score was compared with the CHA_2_DS_2_-VASc using continuous and category-based net reclassification indices (NRIs), integrated discrimination improvement (IDI), c-statistics and decision curve analyses.

**Findings:**

We identified 144,879 anticoagulant naïve patients with new-onset AF between 2007 and 2018 (49.9% women; mean age 72.1 years), of whom 3936 (2.7%) experienced ischaemic stroke during one-year follow-up. Based on both continuous and category-based NRIs, the CHA_2_DS_2_-VA score was inferior to the CHA_2_DS_2_-VASc in the early years (−0.333 (95% CI −0.411 to −0.261) and −0.118 (95% CI −0.137 to −0.099), respectively). However, the differences attenuated over time, and by the end of the study period, the continuous NRI became non-significant (−0.093 (95% CI −0.165 to 0.032)), whereas the category-based NRI reversed in favor of the CHA_2_DS_2_-VA (0.070 (95% CI 0.048–0.087)). The IDI was non-significant in early years (0.0009 (95% CI −0.0024 to 0.0037)), but over time became statistically significant in favor of the CHA_2_DS_2_-VA score (0.0022 (95% CI 0.0001–0.0044)). The Cox models fitted with the CHA_2_DS_2_-VA and the CHA_2_DS_2_-VASc scores exhibited comparable discriminative capability in the beginning of the study (p-value 0.63), but over time marginal differences in favor of the CHA_2_DS_2_-VA score emerged (p-value 0.0002).

**Interpretation:**

In 2007–2008 (when females had higher AF-related stroke risks than males), the CHA_2_DS_2_-VASc score outperformed the CHA_2_DS_2_-VA score, but the initial differences between the scores attenuated over time. By the end of the study period in 2017–2018 (with limited/no sex differences in AF-related stroke), there was marginal superiority for the CHA_2_DS_2_-VA score.

**Funding:**

This work was supported by the 10.13039/100010133Aarne Koskelo Foundation, The Finnish Foundation for Cardiovascular Research, The Finnish State Research funding, and Helsinki and Uusimaa Hospital District research fund.

**Research in context**

**Evidence before this study**

Data from one to two decades ago have shown that females with atrial fibrillation (AF) are at increased risk of ischaemic stroke compared to males. Thus, female sex has been considered as a factor in the evaluation of ischaemic stroke risk in AF, and it has been incorporated into validated risk stratification schemes, such as the widely used CHA_2_DS_2_-VASc score. However, more recent data have shown overall declining ischaemic stroke rates in patients with AF, as well as a decrease in the stroke risk associated with female sex. Therefore, the sex component in the CHA_2_DS_2_-VASc score may no longer provide added predictive value in contemporary populations. We conducted a PubMed search from database inception to April 1, 2024, using the following search terms: ‘Atrial fibrillation’ AND (‘CHA_2_DS_2_-VA’ OR ‘non-sex CHA_2_DS_2_-VASc’) AND ‘Validation’. The few identified validation studies of a non-sex CHA_2_DS_2_-VASc risk score (i.e. CHA_2_DS_2_-VA) have had methodological challenges, particularly regarding sample size, lack of contemporary data, and the consideration of oral anticoagulant therapy.

**Added value of this study**

We evaluated temporal trends of the predictive value of the CHA_2_DS_2_-VA score in relation to the CHA_2_DS_2_-VASc score using nationwide data covering patients with AF from all levels of care in Finland. Throughout the study period from 2007 to 2018, both scores exhibited a relatively good ability in discriminating patients who will experience an ischaemic stroke from those who will not. In 2007–2008, the CHA_2_DS_2_-VASc score outperformed the CHA_2_DS_2_-VA in classifying patients' stroke risk, but the initial differences between the scores attenuated over time, and eventually by the end of the study period in 2017–2018, most used metrics indicated a marginal superiority for the CHA_2_DS_2_-VA score.

**Implications of all the available evidence**

The current study represents the largest validation of the CHA_2_DS_2_-VA score to date, encompassing patients with AF from all levels of care in Finland. However, additional validation studies across diverse patient populations and geographical regions are still warranted. Our findings suggest that the CHA_2_DS_2_-VA score marginally outperforms the CHA_2_DS_2_-VASc in predicting ischemic stroke among contemporary patients with AF. Given this evidence, adopting the CHA_2_DS_2_-VA score could potentially improve accuracy and simplify the assessment of ischemic stroke risk and the need for anticoagulant therapy in patients with AF.

## Introduction

Atrial fibrillation (AF) is the commonest rhythm disorder and confers a substantial mortality and morbidity from stroke, heart failure and dementia, as well as healthcare costs.^1^^,^^2^ Older data from one to two decades ago have shown that females with AF are at increased risk of ischaemic stroke compared to males.^3^ Female patients have also been under-treated with oral anticoagulation (OAC) for stroke prevention compared to males, although this female-male difference has declined in recent years.^4^, ^5^, ^6^, ^7^ Moreover, should female patients with AF present with an ischaemic stroke, these have tended to be of greater severity compared to males.^8^

Given the available evidence, largely from these older studies, female sex was incorporated as a stroke risk factor in the Birmingham algorithm schema in 1996,^9^ which eventually evolved into the CHA_2_DS_2_-VASc risk score.^10^ The latter outperformed the older CHADS_2_ score and was able to further refine stroke risk stratification even if patients were initially categorized as low-risk by the older CHADS_2_ score.^11^ As further evidence evolved, with data showing an age dependence to risk associated with female sex, the sex criterion was proposed as a ‘risk modifier’ rather than a risk factor.^12^ Indeed, in the absence of any non-sex stroke risk factors, there was no difference in ischaemic stroke risk between females and males, but in the presence of 1 or more non-sex stroke risk factors, being female was additive to the ischaemic stroke risk.^13^

Thereafter, contemporary data have shown declining ischaemic stroke rates in relation to AF overall, and in more recent years, there has essentially been no significant difference in the stroke rates between women and men^14^^,^^15^ Hence, a formal evaluation of the predictive value of a non-sex CHA_2_DS_2_-VASc risk score (ie. CHA_2_DS_2_-VA) is warranted with more contemporary data. In this report from the Finnish AntiCoagulation in Atrial Fibrillation (FinACAF) study, we evaluated temporal trends of the predictive, reclassification and discrimination value of the CHA_2_DS_2_-VA score.

## Methods

The FinACAF Study (ClinicalTrials Identifier: NCT04645537; ENCePP Identifier: EUPAS29845) is a nationwide retrospective cohort study that includes all patients documented with AF in Finland from 2004 to 2018.^16^ Patients were identified using all national healthcare registers, including hospitalizations and outpatient specialist visits (HILMO), primary healthcare (AvoHILMO), and the National Reimbursement Register maintained by the Social Insurance Institute (KELA). The cohort inclusion criterion was an International Classification of Diseases, Tenth Revision (ICD-10) diagnosis code of I48, encompassing AF and atrial flutter, collectively referred to as AF, recorded between 2004 and 2018. The exclusion criteria were permanent emigration abroad before December 31, 2018, and age below 20 years at AF diagnosis. The present sub-study was conducted within a cohort of patients diagnosed with incident AF during 2007–2018 that was established in previous studies of the FinACAF cohort.^15^^,^^17^^,^^18^ In this cohort, to include only patients with newly diagnosed AF, a washout period was applied by excluding those with a recorded AF diagnosis or OAC purchases during 2004–2006. Additionally, those with a fulfilled OAC prescription within a year before the first observed AF diagnosis were excluded, as most of these patients may have already had AF diagnosed before.

**Fig. 1:**
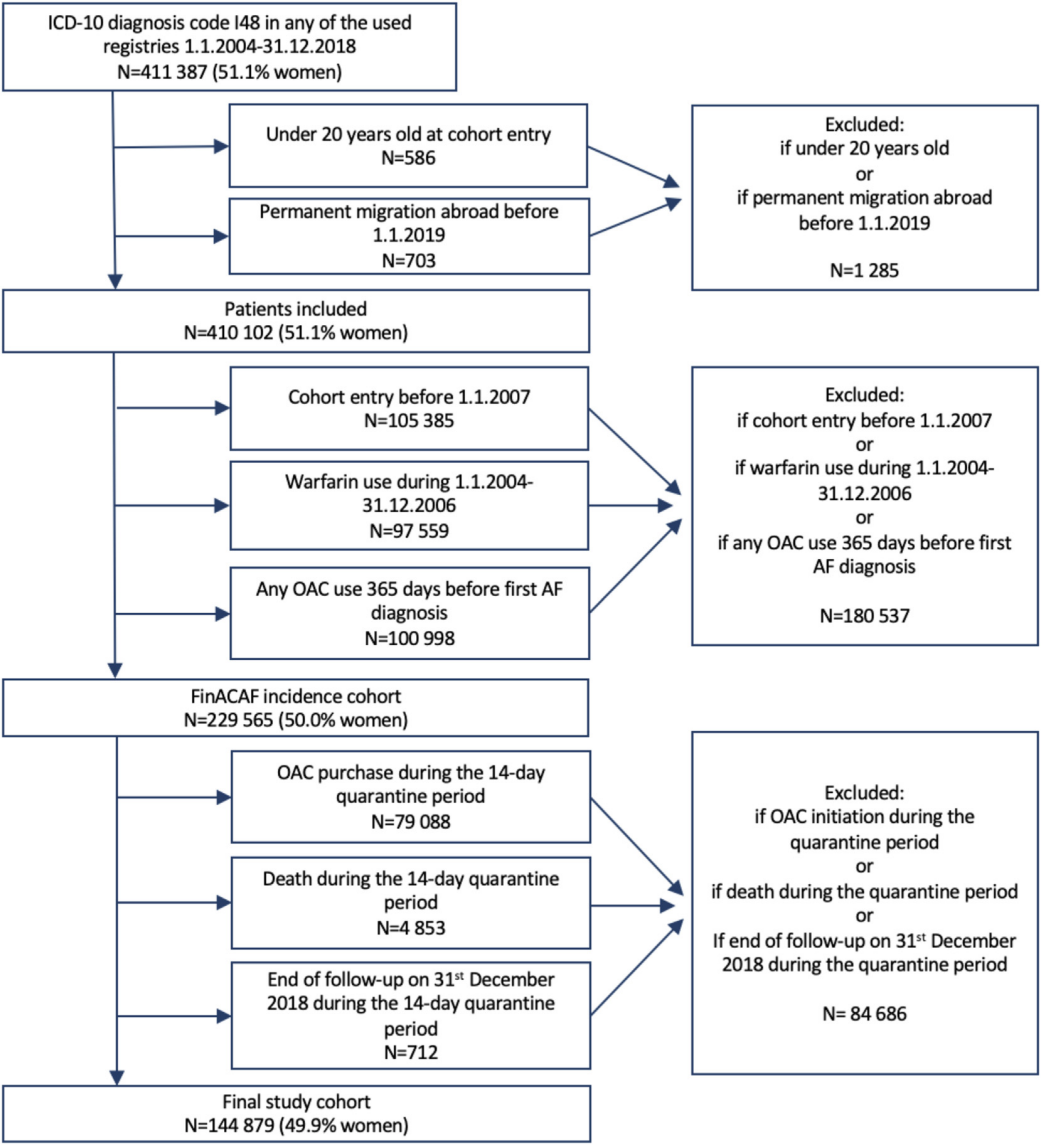
Flow-chart of the patient selection process.

We applied a quarantine period with the follow-up beginning 14 days after the initial AF diagnosis to ensure a stable patient population and to prevent possible double-counting of stroke diagnoses. Stroke events during this blanking period were not considered as outcomes, but were included in the calculation of the baseline risk scores. Patients experiencing death, initiating OAC therapy, or reaching the end of the study period within the quarantine period were excluded. We were interested on the risk of ischaemic stroke in untreated patients and thus concentrated on time without OAC therapy, and therefore, patients were censored when they initiated OAC treatment, marked by the first observed pharmacy purchase of either warfarin or a DOAC (apixaban, dabigatran, edoxaban or rivaroxaban). This method has been recommended for estimating event rates in untreated populations.^19^^,^^20^ Moreover, patients’ risk category may evolve over time due to advancing age and incident comorbidities during a longer follow-up, usually changing from lower to higher categories.^21^^,^^22^ To mitigate this bias in risk group classification, we restricted the follow-up to a maximum of one year after the initial AF diagnosis. This also prevents large variations in follow-up times across the study period, which might affect the interpretation of the stroke trends. Thus, follow-up continued until the first ischaemic stroke event, the first OAC purchase, death, end of study period on December 31st 2018, or a maximum of one year after AF diagnosis, whichever came first. The process of cohort construction is summarized in Fig. 1.

### Risk scores

The baseline CHA_2_DS_2_-VASc score was calculated according to the current guidelines on AF management: congestive heart failure (1 point), hypertension (1 point), age ≥75 years (2 points), diabetes (1 point), history of stroke or TIA (2 points), vascular disease (1 point), age 65–74 years (1 point), sex category (female) (1 point).^12^^,^^23^^,^^24^ The CHA_2_DS_2_-VA score was calculated similarly, but without considering the sex category (Sc). Baseline comorbidity data were obtained from all available nationwide healthcare registers spanning all levels of care, including hospitalizations and outpatient specialist visits, primary healthcare, and pharmacy claims data. The definitions of the comorbidities used in the calculation of the scores are presented in Supplementary Table S1.

### Definition of ischemic stroke

In patients without prior ischaemic stroke, an event was considered to occur on the first date of a recorded I63 or I64 ICD-10 diagnosis code in the hospital care register. In patients with an ischaemic stroke prior to the end of the quarantine period, an ischaemic stroke event was considered to occur on the date of the first new hospitalization with I63 or I64 ICD-10 code as the main diagnosis. The I64 code of unspecified stroke was included in the outcome measure, since it has been shown that 87% of all strokes recorded with ICD-10 code of I64 are ischemic.^25^ Only ischaemic stroke diagnoses from the hospital register were included to ensure that the event of interest was truly major and clinically relevant.

### Study ethics

The study protocol was approved by the Ethics Committee of the Medical Faculty of Helsinki University, Helsinki, Finland (nr. 15/2017), and received research permission from the Helsinki University Hospital (HUS/46/2018). Respective permissions were obtained from the Finnish register holders (KELA 138/522/2018; THL 2101/5.05.00/2018; Population Register Centre VRK/1291/2019-3; Statistics Finland TK-53-1713-18/u1281). Patients’ personal identification numbers were pseudonymized, and the research group received individualized but unidentifiable data. Informed consent was waived due to the retrospective registry nature of the study. The study conforms to the Declaration of Helsinki as revised in 2013.

### Statistical analyses

The study period from 2007 to 2018 was divided into two-year periods. Stroke rates with 95% confidence intervals in each calendar year period according to the risk score points were estimated with Poisson regression. Considering the limitations of any single risk prediction measure *per se*, we used a set of metrics that were considered appropriate for the current research question of comparing the two risk prediction scores.^26^ The risk scores were fitted as categorical variables in Cox regression and the discriminative capacity of the scores was quantified with the Harrel's c-statistics. The cause-specific Cox proportional hazards models considered death and OAC initiation as competing events. The models were compared using the non-nested likelihood ratio test.^27^ The proportional hazards assumption was tested by plotting Schoenfeld residuals and no violation was observed in any of the calendar year periods. Moreover, receiver operating characteristics curves for ischaemic stroke at one-year follow-up were computed to summarize the sensitivity and specificity of the risk scores. To guide clinical decision making regarding OAC therapy, an optimal stroke risk scoring system should categorize patients into low, moderate, and high-risk groups. According to current guidelines, OAC therapy is typically not recommended for low-risk patients, should be considered for moderate-risk patients, and is recommended for high-risk patients.^12^^,^^23^^,^^24^ Hence, to assess the efficacy of risk scores in stratifying patients into these categories, we computed category-based net reclassification index (NRI) with annual risk thresholds of 1% and 2% between the low, moderate and high-risk groups, aligning with the risk stratification of the most recent American guidelines on AF management.^23^ The category-based NRI has a range from −2 to +2 and it quantifies the net proportion of patients experiencing an event who are upclassified to a higher risk category plus the net proportion of patients without an event who are downclassified.^28^ Additionally, continuous NRI and integrated discrimination improvement (IDI) were computed to compare the scores without the somewhat artificial risk categorization. The continuous (category-free) NRI quantifies the net proportion of patients with events predicted with a higher risk and patients without events predicted with a lower risk compared to the reference model. It does not consider the magnitude of the predicted risk difference, and focuses only on the upward and downward risk movement for patients with and without events.^26^ On the other hand, the IDI summarizes the difference in the means of predicted risks for patients with and without events, thereby better capturing the magnitude of the discriminatory disparities between the models.^26^ The 95% confidence intervals for the NRIs and IDI were obtained by bootstrapping.^26^^,^^29^ Finally, we conducted decision curve analyses for each calendar year interval to depict the net benefit of the risk scores in identifying patients with ischaemic stroke at a range of annual risk thresholds. Event probabilities were derived from the aforementioned Cox regression models.^30^ Chi-square test and analysis of variance were used to compare baseline variables. A p-value of 0.05 was used as the threshold for statistical significance. Statistical analyses were performed with R version 4.0.5 (R Core Team, Vienna, Austria; https://www.R-project.org).

### Sensitivity analyses

To alleviate potential variability in the score metrics resulting from the smaller number of patient-years and events when dividing the study period into two-year intervals, we also conducted analyses with the study period divided into four-year intervals. Moreover, while we primarily focused on the initial risk stratification in patients with new-onset AF to mitigate the impact of dynamic changes in stroke risk due to aging and new comorbidities, and thus analyzed only the first year of follow-up after the AF diagnosis, we also assessed differences in the risk scores when the follow-up was extended to a maximum of two years.^21^^,^^22^

### Role of the funding source

The funders had no role in the study design, any aspect of conducting the study, interpretation of findings, or the decision to submit the paper for publication.

## Results

We identified 144,879 OAC naïve patients with new-onset AF between 2007 and 2018 (49.9% women; mean age 72.1 years, SD 14.3). Baseline age and the prevalence of most comorbidity components of the risk scores increased over the study period, leading to a rise in the mean of both scores (Table 1). Use of OAC therapy increased during the study period, so that the follow-up ended to OAC initiation in 20.2% of patients in 2007–2008 and in 68.2% of patients in 2017–2018. This resulted in a decrease in the total patient-years of follow-up towards the end of the study period (Table 2).

**Table 1:** Baseline characteristics of the study cohort according to the calendar year intervals.

	2007–2008	2009–2010	2011–2012	2013–2014	2015–2016	2017–2018
n	21,892	20,783	20,650	20,339	26,611	34,559
Mean age (years)	71.1	71.0	71.4	70.9	72.4	74.2
Mean CHA_2_DS_2_-VASc	3.1	3.2	3.3	3.3	3.5	3.7
Mean CHA_2_DS_2_-VA	2.6	2.7	2.8	2.8	3.0	3.2
Congestive Heart failure	15.2	16.5	17.0	17.4	15.9	16.2
Hypertension	65.9	69.2	70.9	71.9	75.9	79.2
Age 65–74	20.4	21.4	22.1	24.1	27.4	28.3
Diabetes	15.7	16.9	18.6	20.4	23.5	25.6
Prior ischaemic stroke or TIA	19.6	19.4	20.7	19.8	20.3	19.7
Any vascular disease	26.1	26.9	28.1	27.6	27.3	29.4
Age 75 or more	47.7	46.7	47.4	45.0	48.2	52.8
Female sex	51.5	51.5	49.8	48.1	48.8	49.7
CHA_2_DS_2_-VASc = 0	10.2	9.1	8.7	8.9	6.6	4.3
CHA_2_DS_2_-VASc = 1	14.0	14.0	13.6	13.9	10.8	8.6
CHA_2_DS_2_-VASc = 2	15.8	16.1	15.3	15.4	14.9	13.9
CHA_2_DS_2_-VASc = 3	17.0	16.5	16.4	15.9	18.0	19.1
CHA_2_DS_2_-VASc = 4	19.3	19.0	18.4	17.2	19.7	21.6
CHA_2_DS_2_-VASc = 5	12.7	12.8	13.1	13.4	14.6	15.5
CHA_2_DS_2_-VASc = 6	7.3	7.8	8.7	8.9	9.2	10.1
CHA_2_DS_2_-VASc = 7	2.9	3.5	4.2	4.3	4.4	4.9
CHA_2_DS_2_-VASc = 8	0.8	1.1	1.4	1.7	1.6	1.5
CHA_2_DS_2_-VASc = 9	0.2	0.2	0.3	0.3	0.3	0.4
CHA_2_DS_2_-VA = 0	13.7	12.7	12.1	12.2	9.0	5.8
CHA_2_DS_2_-VA = 1	17.3	17.6	16.6	17.0	13.6	11.7
CHA_2_DS_2_-VA = 2	17.6	17.0	16.7	16.3	17.8	17.6
CHA_2_DS_2_-VA = 3	22.1	21.2	20.3	19.1	21.8	23.8
CHA_2_DS_2_-VA = 4	15.4	15.6	15.9	15.4	17.3	18.4
CHA_2_DS_2_-VA = 5	8.9	9.6	10.8	11.5	11.6	12.8
CHA_2_DS_2_-VA = 6	3.7	4.5	5.3	5.6	5.8	6.7
CHA_2_DS_2_-VA = 7	1.2	1.6	2.0	2.3	2.4	2.5
CHA_2_DS_2_-VA = 8	0.2	0.3	0.5	0.5	0.6	0.6

Values depict proportions (%) unless otherwise specified. All differences between calendar year intervals p < 0.001.

Abbreviations: TIA, transient ischemic attack, CHA_2_DS_2_-VA(Sc), congestive heart failure (1 point), hypertension (1 point), age ≥75 years (2 points), diabetes (1 point), history of stroke or TIA (2 points), vascular disease (1 point), age 65–74 years (1 point), sex category (female) (1 point).

**Table 2:** Trends of the ischemic stroke rates according to the risk score points.

	2007–2008	2009–2010	2011–2012	2013–2014	2015–2016	2017–2018
Patient-years	12,053	14,917	13,987	13,351	12,519	11,460
Events (n)	677	716	694	654	626	596
**Incidence rates (per 100 patient years**)						
CHA_2_DS_2_-VASc points						
0	0.4 (0.1–0.9)	0.5 (0.2–0.9)	0.4 (0.1–0.8)	0.5 (0.2–1.0)	0.7 (0.3–1.2)	0.6 (0.3–1.2)
1	0.7 (0.4–1.2)	0.9 (0.6–1.3)	0.5 (0.3–0.9)	0.6 (0.3–1.0)	1.2 (0.8–1.7)	1.1 (0.6–1.6)
2	2.3 (1.7–3.0)	1.6 (1.2–2.2)	2.2 (1.7–2.9)	1.7 (1.2–2.3)	1.9 (1.9–3.4)	2.6 (1.9–3.4)
3	3.9 (3.1–4.9)	3.5 (2.8–4.3)	3.3 (2.6–4.1)	3.6 (2.8–4.5)	3.8 (3.0–4.8)	4.1 (3.2–5.1)
4	6.8 (5.7–7.9)	5.8 (4.9–6.8)	7.3 (6.3–8.5)	6.6 (5.5–7.7)	6.0 (4.9–7.2)	6.4 (5.3–7.6)
5	12.9 (11.0–15.0)	9.8 (8.3–11.5)	10.0 (8.4–11.7)	9.7 (8.2–11.5)	9.6 (8.0–11.5)	7.6 (6.2–9.3)
6	21.0 (17.6–24.8)	16.8 (14.1–19.8)	15.0 (12.5–17.8)	15.2 (12.5–17.8)	14.6 (12.2–17.4)	13.3 (10.9–16.6)
7	28.2 (22.0–35.7)	21.8 (17.1–27.3)	15.3 (11.7–19.7)	18.6 (14.6–23.2)	20.6 (16.3–25.7)	14.8 (11.3–19.0)
8	21.5 (11.8–36.1)	20.6 (12.9–31.2)	17.6 (11.3–26.2)	17.2 (11.2–25.2)	16.9 (10.6–25.5)	16.3 (10.4–24.2)
9	0 (0–23.1)	47.0 (22.6–86.5)	24.6 (9.0–53.6)	20.9 (6.8–48.8)	24.8 (10.0–51.1)	15.9 (5.8–34.7)
CHA_2_DS_2_-VA points						
0	0.3 (0.1–0.7)	0.5 (0.3–0.9)	0.4 (0.2–0.7)	0.4 (0.2–0.7)	0.6 (0.3–1.0)	0.5 (0.2–1.0)
1	0.9 (0.6–1.4)	0.9 (0.6–1.3)	1.0 (0.7–1.5)	0.8 (0.5–1.2)	1.4 (1.0–1.9)	1.5 (1.0–2.1)
2	3.4 (2.7–4.3)	2.3 (1.8–3.0)	2.6 (2.0–3.4)	2.4 (1.8–3.2)	2.4 (1.7–3.1)	2.9 (2.2–3.8)
3	5.8 (4.9–6.8)	4.9 (4.1–5.7)	5.7 (4.8–6.6)	5.1 (4.3–6.1)	4.8 (4.0–5.8)	5.1 (4.2–6.2)
4	9.8 (8.3–11.5)	7.7 (6.5–9.0)	8.4 (7.1–9.8)	8.5 (7.1–10.0)	8.3 (7.0–9.9)	7.3 (6.0–8.8)
5	20.9 (17.8–24.3)	15.5 (13.2–18.1)	14.5 (12.4–17.0)	13.1 (11.1–15.3)	11.9 (9.9–14.2)	11.2 (9.3–13.4)
6	25.1 (19.8–31.2)	22.7 (18.5–27.6)	15.4 (12.2–19.2)	19.5 (16.0–23.7)	21.0 (17.3–25.3)	15.5 (12.4–19.2)
7	22.6 (14.3–33.9)	22.3 (15.5–31.0)	16.5 (11.3–23.1)	16.5 (11.6–22.9)	19.5 (13.8–26.8)	14.7 (10.1–20.8)
8	9.4 (1.1–33.9)	35.5 (17.7–63.5)	25.8 (12.4–47.5)	18.9 (8.1–37.1)	17.5 (8.0–33.2)	19.5 (10.1–34.0)

Abbreviations: CHA_2_DS_2_-VA(Sc), congestive heart failure (1 point), hypertension (1 point), age ≥75 years (2 points), diabetes (1 point), history of stroke or transient ischemic attack (2 points), vascular disease (1 point), age 65–74 years (1 point), sex category (female) (1 point). 95% confidence intervals in parenthesis.

Overall, 3936 (2.7%) patients experienced an ischaemic stroke during the one-year follow-up from AF diagnosis. The rate of ischaemic stroke increased gradually with rising CHA_2_DS_2_-VA and CHA_2_DS_2_-VASc scores, although some variability was observed in the highest score point categories. The ischaemic stroke rates showed an overall declining pattern among patients with higher risk score points (Table 2). The rate trends were similar, but with less variability, when the study period was divided into four-year intervals (Supplementary Table S2).

### Comparisons of the CHA_2_DS_2_-VA and CHA_2_DS_2_-VASc scores

**Fig. 2:**
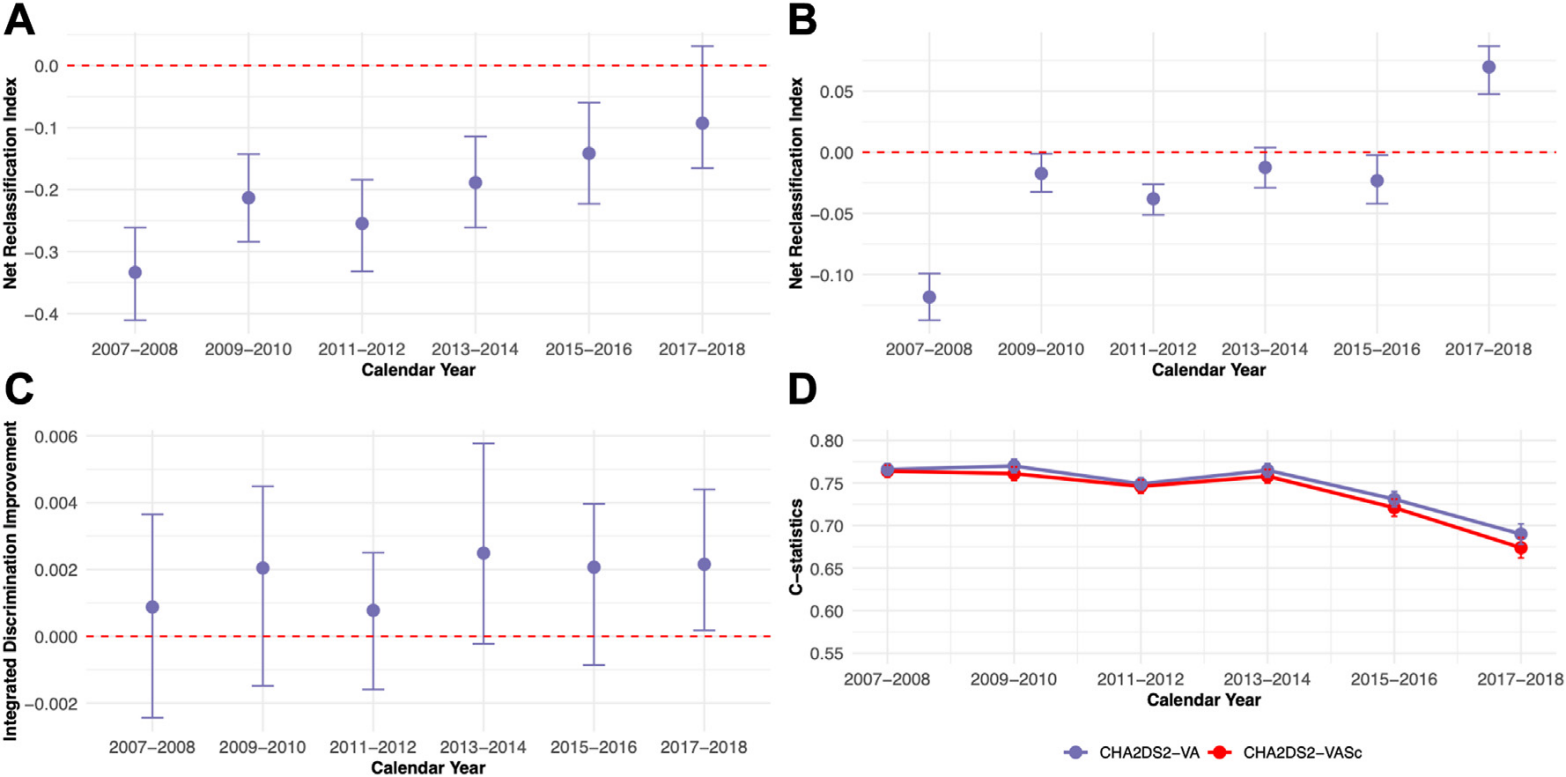
Temporal trends of continuous net reclassification index (panel A), category-based net reclassification index (panel B), integrated discrimination improvement (panel C) and c-statistics of the proportional hazards models (panel D). Footnote: In panels A–C, the red horizontal dashed line at zero level represents the CHA_2_DS_2_-VASc score as the reference. Values below this line are in favor of the CHA_2_DS_2_-VASc score, while values above favor the CHA_2_DS_2_-VA score. Error bars represent 95% confidence intervals. 1% and 2% thresholds were used to separate low-, moderate-, and high-risk categories in the category-based net reclassification index. p-values for differences in the proportional hazards models 0.21, 0.0009, 0.63, 0.032, 0.0011 and 0.0044 for calendar year periods 2007–2008, 2009–2010, 2011–2012, 2013–2014, 2015–2016 and 2017–2018, respectively.

According to both the continuous and category-based NRI, the CHA_2_DS_2_-VA score was inferior to the CHA_2_DS_2_-VASc in the early years of the study period; however, the difference in the continuous NRI attenuated and became non-significant over time (Fig. 2, panel A). The category-based NRI became similarly first non-significant and eventually reversed in favor of the CHA_2_DS_2_-VA score by 2017–2018 (Fig. 2, panel B). Based on the IDI, the risk scores appeared to have a similar discriminative capacity in the first calendar year intervals, but by end of the study period, the IDI values increased slightly and became statistically significant in favor of the CHA_2_DS_2_-VA score (Fig. 2, panel C).

The c-statistics exhibited a slight decrease over time, yet consistently indicating a fair discriminative ability throughout the study period for both scores. The Cox models fitted with the CHA_2_DS_2_-VA and the CHA_2_DS_2_-VASc exhibited comparable discriminative ability in the periods 2007–2008 and 2011–2012 (p-values for difference 0.21 and 0.63, respectively). However, in all other intervals, the model fitted with the CHA_2_DS_2_-VA score was superior to that of the CHA_2_DS_2_-VASc (p-values <0.05; Fig. 2, panel D). In sensitivity analysis, the trends patterns of the c-statistics, the IDI, and the NRIs were comparable also when the study period was divided into four-year intervals (Supplementary Figure S1). The patterns of these metrics were also similar when the follow-up was extended to two years, with the category-based NRI and the c-statistics favoring the CHA_2_DS_2_-VA score at the end of the study period (Supplementary Figure S2).

**Fig. 3:**
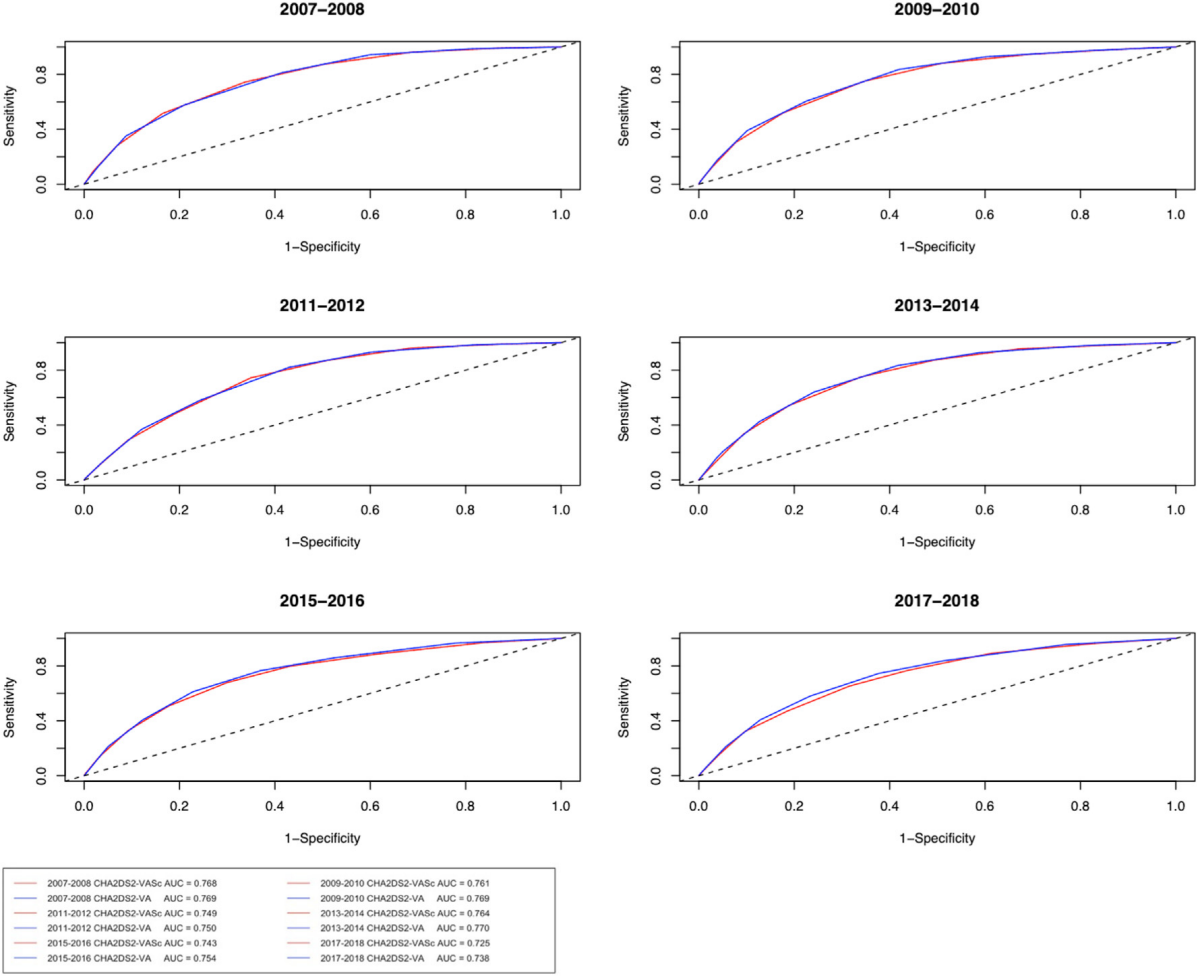
Receiver operating characteristic curves at one-year follow-up according to the calendar year intervals for CHA_2_DS_2_-VA (blue line) and CHA_2_DS_2_-VASc (red line) scores. Footnote: p-values for area under curve differences between the scores 0.63, 0.040, 0.86, 0.022, 0.0007 and 0.0002 for calendar year periods 2007–2008, 2009–2010, 2011–2012, 2013–2014, 2015–2016 and 2017–2018, respectively.

**Fig. 4:**
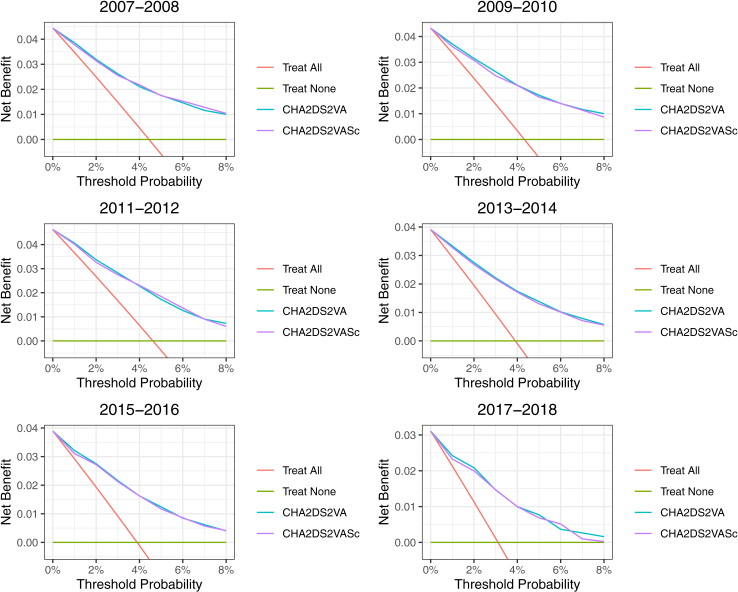
Decision curve analyses in different calendar year intervals. Footnote: Net number of true positives detected using different scores compared with no model at a range of threshold probabilities.

Correspondingly, the receiver operating characteristics curves of the scores at one year follow-up largely overlapped in most calendar year periods, yet towards the end of the study period, small but statistically significant differences were observed in the areas under curves in favor of the CHA_2_DS_2_-VA score (Fig. 3). In the decision curve analyses, the curves for the risk scores essentially overlapped, indicating a similar net benefit in detecting patients who will experience an ischaemic stroke when compared to a situation without a risk stratification system. However, in the last two calendar year periods, a subtle difference emerged in favor of the CHA_2_DS_2_-VA score in annual risk threshold levels below 2% (Fig. 4).

## Discussion

In this formal evaluation of the predictive value of the non-sex CHA_2_DS_2_-VASc risk score (i.e. CHA_2_DS_2_-VA) in relation to the CHA_2_DS_2_-VASc score, our principal findings are as follows: (1) Throughout the study period from 2007 to 2018, both scores exhibited a relatively good ability in discriminating patients who will experience an ischaemic stroke from those who will not; (2) In 2007–2008 (when females had higher AF-related stroke risks than males), the CHA_2_DS_2_-VASc score outperformed the CHA_2_DS_2_-VA in classifying patients' stroke risk; and (3) The initial differences between the scores attenuated over time, and eventually by the end of the study period in 2017–2018 (with limited/no sex differences in AF-related stroke), most used metrics indicated a marginal superiority for the CHA_2_DS_2_-VA score.

There are many stroke risk factors in patients with AF, but the most common and validated ones have been used to formulate risk stratification scores to aid decision making in everyday clinical practice. Nonetheless, all clinical risk scores are simplifications, have many limitations, and typically possess only a modest predictive value for identifying high-risk patients. While more detailed risk scores, such as the ATRIA score, have demonstrated slightly superior stroke prediction capabilities, their adoption into bed-side clinical practice has been impeded by their complexity.^31^^,^^32^ Moreover, stroke risk is dynamic, not static, changing with ageing and incident comorbidities.^22^ Also, simple clinical risk scores based on factors derived at baseline do not account for the competing risk of death, nor the decelerating treatment benefit over time, especially when life expectancy is short.^33^ Finally, statistical significance in predicting stroke rates is not the same as clinical significance of absolute risks, which needs to be balanced with practical application.

The female sex (Sc) criterion features in the CHA_2_DS_2_-VASc score as older studies suggested an excess in ischaemic stroke risk in female patients compared to males.^34^ For example, in a systematic review and meta-analysis, Emdin et al. demonstrated that in the presence of AF, there was a higher risk of stroke in women compared with men (ratio of relative risk 1.99, 95% CI 1.46–2.71).^35^ Likewise, the meta-analysis of 17 studies by Wagstaff et al. revealed a 1.31-fold (95% CI 1.18–1.46) elevated risk of stroke in women with AF.^3^ In the ORBIT-AF Registry, women with AF had more symptoms and worse quality of life, and despite their lower risk-adjusted all-cause and cardiovascular mortality, women had higher stroke rates (adjusted HR 1.39, 95% CI 1.05–1.84).^36^ Hence, female sex was incorporated as a factor into the CHA_2_DS_2_-VASc risk score, along with vascular disease and added points for age, i.e. 1 point for age 65–74 and 2 points for age ≥75.^10^

Nonetheless, studies on Danish registries thereafter reported that in the absence of non-sex stroke risk factors, there was no significant difference in ischaemic stroke risk between females and males, and that being female was additive to ischaemic stroke risk only in the presence of 1 or more non-sex stroke risk factors.^13^ However, data from older observational cohort studies may not reflect contemporary stroke rates, given a general decline in stroke risk in more recent years with better detection and management of risk factors.^37^^,^^38^ We have also reported similarly declining overall stroke rates in patients with AF in Finland, and importantly, in more recent years, there was no difference in stroke risk between women and men.^14^^,^^15^

In the present comprehensive evaluation of the temporal trends in the predictive value of the non-sex CHA_2_DS_2_-VASc risk score (ie. CHA_2_DS_2_-VA), we show that the CHA_2_DS_2_-VASc score was broadly similar or superior to the CHA_2_DS_2_-VA in stroke prediction in 2007–2008. Both the continuous and the category-based NRIs indicated a more accurate risk classification with the CHA_2_DS_2_-VASc at the start of the study period, whereas at this point all other metrics showed comparable performance for the scores. However, over time these initial differences attenuated, and by the end of the study period (when there were limited/no sex differences in AF-related stroke), subtle differences emerged in favor of the CHA_2_DS_2_-VA score. Indeed, only the continuous NRI was non-significant at the last calendar year period, while other metrics showed a marginal yet statistically significant superiority for the CHA_2_DS_2_-VA score. The patterns of the continuous and category-based NRIs were somewhat different, with a slower and more linear change observed in the continuous NRI, which measures reclassification across the risk spectrum, likely reflecting the previously reported attenuation of sex-related differences in stroke rates even in high-risk patients.^14^ Importantly, in the last calendar year period, the category-based NRI showed that the CHA_2_DS_2_-VA was superior in classifying patients to low-, moderate, and high-risk categories. This finding was supported by the decision curve analyses, which in the end of the study favored the use of the CHA_2_DS_2_-VA particularly at the risk threshold range of 1–2%, a crucial range regarding the harms and benefits of OAC therapy.

Of note, reliance solely on the c-statistics, or in fact on any single metric of risk prediction value, has many limitations.^26^^,^^39^ All clinical factor-based scores perform only modestly, with c-statistics approximately 0.65, so our findings are unsurprising regarding the limited differences in c-statistics between the CHA_2_DS_2_-VASc and CHA_2_DS_2_-VA scores, and prior studies that only focus on this metric do not convey the full picture. Therefore, our inclusion of the trends in reclassification and discrimination metrics, along with the decision curve analyses, provides a comprehensive and formal evaluation of the predictive value of the CHA_2_DS_2_-VA score, extending also to more contemporary patient populations.

However, it is still important to note that the observed differences were overall only marginal, and that the c-statistics indicated a relatively fair discriminative ability across the study period for both scores. Thus, the mere statistical superiority at the end of study does not automatically guarantee tangible real-world benefits in opting for the CHA_2_DS_2_-VA score over CHA_2_DS_2_-VASc in clinical decision-making on OAC therapy. Nonetheless, current AF management guidelines recommend different CHA_2_DS_2_-VASc point thresholds in women and men for the initiation of OAC therapy.^12^^,^^23^ In fact, in current real-life patient care, the point from female sex does not significantly impact treatment decisions and serves more as a reminder of the elevated stroke risk in women who already have other stroke risk factors. Therefore, the current use of the CHA_2_DS_2_-VASc score for anticoagulation decision-making already resembles the use of the CHA_2_DS_2_-VA score. Given the previously reported decline in stroke risk associated with female sex and the current findings regarding the superiority of the CHA_2_DS_2_-VA score among the more contemporary patient cohorts, opting for the CHA_2_DS_2_-VA might both improve accuracy and simplify the evaluation of stroke risk and the need for OAC therapy in clinical practice.

The most important factor underlying the observed trends in the score differences is indeed most likely the previously reported decrease in the stroke risk associated with female sex in the FinACAF study cohort.^14^ However, some other factors may also be at play. Stroke risk among patients with high score points decreased during the study period, which is in line with previous reports of overall decreasing stroke rates.^38^ This decline may impact the scores' predictive capabilities and could be linked to earlier detection of AF, as well as to changes in the diagnosis and management of other stroke risk factors. There also appeared to be a rising trend in the proportion of patients with higher risk scores at baseline. In addition to increasing age, this may signal improved detection of comorbidities, possibly leading to the recategorization of lower-risk patients into higher risk categories. Such recategorization may also partly explain the observed changes in risk score measures, the declining c-statistics towards the end of the study period, and the declining stroke rates in higher-risk score categories. Nevertheless, our previous findings indicate that the independent impact of diabetes and hypertension on ischemic stroke risk have actually remained static during the same study period.^40^^,^^41^

Prior validations of the CHA_2_DS_2_-VA score have had methodological challenges. For example, Maeda et al. used a modest sized Japanese cohort of 9733 patients whereby the c-statistics were similar for the CHA_2_DS_2_-VASc score and the CHA_2_DS_2_-VA score (c-statistics with 95% CIs 0.70 (0.65–0.74) and 0.70 (0.66–0.75), respectively, p = 0.48).^42^ However, they only studied patients who were never prescribed anticoagulation agents during follow-up, leading to ‘conditioning on the future’, which biases the observed event rates. In the J-RHYTHM registry, the c-statistics in the CHADS2, *m*CHA_2_DS_2_-VASc, and *m*CHA_2_DS_2_-VA scores were 0.58 (95% CI 0.45–0.70), 0.63 (95% CI 0.48–0.76), and 0.63 (95% CI 0.49–0.75), respectively.^43^ While their cohort was non-anticoagulated at baseline, treatment with warfarin was initiated in 234 (23%) patients during the follow-up period with no time-in-therapeutic-range data, and only modest differences were seen in the c-indexes which were all below 0.7. The largest published study used Korean nationwide data and reported comparable predictive abilities for of the CHA_2_DS_2_-VA and CHA_2_DS_2_-VASc scores, but this study was subsequently retracted.^44^ Despite the limited published validation data, in 2018, the guidelines of the National Heart Foundation of Australia and the Cardiac Society of Australia and New Zealand recommended risk stratification based on the CHA_2_DS_2_-VA scores of 0 (low), 1 (intermediate) and ≥2 (high).^45^

There have been numerous CHA_2_DS_2_-VASc validations in different cohorts globally, and virtually all these studies have shown broadly similar c-indexes (approximately 0.65), as would be expected for all clinical factor based risk scores performed in AF (CHADS_2_, CHA_2_DS_2_-VASc, etc) and non-AF settings. For the CHA_2_DS_2_-VA score, the c-indexes are again largely the same, as seen also in the abovementioned smaller prior studies. However, the major novelty in our paper is the time trends analysis—in the early years, when females with AF had higher ischaemic stroke risk than males, using the CHA_2_DS_2_-VASc score was reasonable, and it outperformed the CHA_2_DS_2_-VA score. In more recent years, where there have been hardly any sex differences in AF-related stroke, the present study now robustly validates that the CHA_2_DS_2_-VA score had essentially no difference to the CHA_2_DS_2_-VASc in a non-anticoagulated cohort, and that the CHA_2_DS_2_-VA score may arguably even be, at least statistically, slightly superior for risk stratification.

Notwithstanding the large nationwide data and unique coverage of all levels of care, the limitations of our study need to be acknowledged, the most important of which are inherent to the observational registry-based study design. Indeed, information bias may affect the detection of patients with AF, the identification of comorbidities, as well as the accuracy of the outcome events. However, the utilized hospital register has been well-validated, showing relatively high diagnostic accuracy, particularly regarding cardiovascular diseases.^46^ Additionally, selection bias may be present in the formation of the study cohort, although the methods in selecting patients with incident AF, the use of a quarantine period at the start of the follow-up, and censoring for patients who initiate OAC treatment align with prior robust studies in this field and recommendations in estimating stroke rates in OAC naïve patient populations.^20^^,^^47^

Moreover, use of OAC therapy in Finland has increased considerably during our study period, which might affect the results, but aligning with prior validation studies on stroke risk scores, we concentrated on estimating event rates, i.e. events per time. Thus, while the increasing number of censored patients due to OAC initiation might affect the observed cumulative incidence of stroke, OAC initiation has less impact on the observed event rates. Also, as we have previously reported, there have been only marginal gender disparities in OAC use in Finland during the same study period.^7^ Additionally, we focused on the initial risk stratification in patients with new-onset AF, and did not consider dynamic changes in stroke risk related to aging, incident comorbidities or disease severity (for example blood pressure control). Furthermore, we lacked data on the subtype of AF. Taken together, while all these aspects may impact the absolute stroke rate figures, we assume their influence on the actual comparison of the risk scores to be minor. Finally, while our study covers patients with AF from all levels of care in Finland until 2018, additional validation studies are needed across diverse patient populations and geographical regions, as well as with data from even more recent years.

### Conclusion

In this formal evaluation of the non-sex CHA_2_DS_2_-VASc risk score (i.e. CHA_2_DS_2_-VA) in patients with AF, we show that amidst declining stroke rates, the CHA_2_DS_2_-VA score was marginally superior to the CHA_2_DS_2_-VASc score in stroke risk stratification among more contemporary patients with AF. Opting for the CHA_2_DS_2_-VA could both improve accuracy and simplify the evaluation of stroke risk and decision-making for OAC therapy in contemporary clinical practice.

## Contributors

KT and GYHL contributed to the conceptualization, investigation, and methodology of the study. KT also handled data curation, formal analysis, visualization, and wrote the original draft. GYHL provided supervision and contributed to the original draft writing. KT and GYHL contributed equally to this work. KEJA, OH, JHau, JHar, JP, PM, MLi, and MLe were involved in conceptualization and the review and editing of the manuscript. KEJA also participated in investigation, methodology, project administration, and supervision. OH additionally handled data curation, formal analysis, and methodology. KT and OH have verified the data. JH took part in methodology, supervision, and project administration. JP and JHar also contributed resources and supervision. MLe was responsible for funding acquisition, project administration, resources, and supervision. All authors had final responsibility for the decision to submit for publication.

## Data sharing statement

Because of the sensitive nature of the data collected for this study, requests to access the dataset from qualified researchers trained in human subject confidentiality protocols may be sent to the Finnish national register holders (KELA, Finnish Institute for Health and Welfare, Population Register Center and Tax Register) through Findata (https://findata.fi/en/).

## Declaration of interests

KT: Research Grants: The Finnish Foundation for Cardiovascular Research, Aarne and Aili Turunen Foundation and the Finnish State Research Funding GYHL: None. OH: none. JP: Speaker: Bayer, Boehringer-Ingelheim, BMS-Pfizer, Abbott; Advisory board: Portola, Novo Nordisk, Herantis Pharma; Visiting editor: Terve Media; Stock ownership: Vital Signum. PM: Consultant: Roche, BMS-Pfizer-alliance, Novartis Finland, Boehringer Ingelheim, MSD Finland. JHau: Consultant: Research Janssen R&D; Speaker: Bayer Finland. MLi: Speaker: BMS-Pfizer-alliance, Bayer, Boehringer-Ingelheim. JHar: Research grants: The Finnish Foundation for Cardiovascular Research, EU Horizon 2020, EU FP7. Advisory Board Member: BMS-Pfizer-alliance, Novo Nordisk, Amgen. Speaker: Cardiome, Bayer. KEJA: Research grants: The Finnish Foundation for Cardiovascular Research; Speaker: Bayer, Pfizer and Boehringer-Ingelheim. Member in the advisory boards: Bayer, Pfizer and AstraZeneca. MLe: Consultant: BMS-Pfizer-alliance, Bayer, Boehringer-Ingelheim, and MSD; Speaker: BMS-Pfizer-alliance, Bayer, Boehringer Ingelheim, MSD, Terve Media and Orion Pharma. Research grants: Aarne Koskelo Foundation, The Finnish Foundation for Cardiovascular Research, and Helsinki and Uusimaa Hospital District research fund, Boehringer-Ingelheim.
