# Supplementary material for: Comparing CHA2DS2-VA and CHA2DS2-VASc scores for stroke risk stratification in patients with atrial fibrillation: a temporal trends analysis from the retrospective Finnish AntiCoagulation in Atrial Fibrillation (FinACAF) cohort
Source: Lancet Reg Health Eur. 2024 Jun 10;43:100967. doi: 10.1016/j.lanepe.2024.100967 (PMC11337097; doi:10.1016/j.lanepe.2024.100967)
Supplement: Supplementary Figures and Tables [file mmc1.docx]

**Supplementary Material**

**Supplementary Table 1.** Definitions of the comorbidities

**Supplementary Table 2.** Ischemic stroke rates according to the risk score points in four-year intervals.

**Supplementary Figure 1.** Results of the sensitivity analyses with the cohort divided into four-year intervals. Temporal trends of continuous net reclassification index (panel A), category-based net reclassification index (panel B), integrated discrimination improvement (panel C) and c-statistics of the proportional hazards models (panel D).

**Supplementary Figure 2.** Results of the sensitivity analyses with a two-year follow-up. Temporal trends of continuous net reclassification index (panel A), category-based net reclassification index (panel B), integrated discrimination improvement (panel C) and c-statistics of the proportional hazards models (panel D).

**Supplementary Table 1**. Definitions of the comorbidities

|  | ICD-10 | ICPC-2 | Reimbursement code | ATC code |
| --- | --- | --- | --- | --- |
| Any vascular disease | I20-I25, I65-I66, I67.2, I70 | K74, K75, K76, K91, K92 | 206 |  |
| Diabetes | E10-E14 | T89, T90 | 103, 215 | A10 |
| Heart failure | I50, I11.0, I13.0, I13.2 | K77 | 201 |  |
| Hypertension | I10-I15 | K85, K86, K87 | 205 | C03A, C03B, C03DB, C03EA, C07A, C08CA, C08D, C09 |
| Previous stroke | I63, I64, I69.3-I69.8 | K90 |  |  |
| Transient ischemic attack | G45 | K89 |  |  |
| Abbreviations: ATC, anatomic therapeutic chemical; ICD-10, International Classification of Diseases, Tenth Revision; ICPC-2, International Classification of Primary Care, Second Edition | | | | |

**Supplementary Table 2.** Ischemic stroke rates according to the risk score points in four-year intervals.

|  | **2007-2010** | **2011-2014** | **2015-2018** |
| --- | --- | --- | --- |
| Patient-years | 26 970 | 27 350 | 23 979 |
| Events (n) | 1 393 | 1 348 | 1 195 |
| **Incidence rates (per 100 patient years**) | |  |  |
| CHA_2_DS_2_-VASc points | |  |  |
| 0 | 0.4 (0.2-0.7) | 0.4 (0.2-0.7) | 0.6 (0.4-1.0) |
| 1 | 0.8 (0.6-1.1) | 0.6 (0.4-0.8) | 1.1 (0.8-1.5) |
| 2 | 1.9 (1.5-2.3) | 2.0 (1.6-2.4) | 2.2 (1.8-2.8) |
| 3 | 3.7 (3.1-4.3) | 3.4 (2.9-4.0) | 3.9 (3.3-4.6) |
| 4 | 6.2 (5.5-7.0) | 6.9 (6.2-7.8) | 6.2 (5.4-7.0) |
| 5 | 11.2 (10.0-12.5) | 9.8 (8.7-11.0) | 8.6 (7.6-9.8) |
| 6 | 18.6 (16.5-20.9) | 15.1 (13.3-20.9) | 13.9 (12.2-15.8) |
| 7 | 24.5 (20.7-28.8) | 17.0 (14.2-20.1) | 17.6 (14.8-20.8) |
| 8 | 21.0 (14.7-29.0) | 17.4 (12.9-22.9) | 16.6 (12.1-22.1) |
| 9 | 26.8 (12.9-49.4) | 22.8 (11.4-40.8) | 19.7 (10.5-33.8) |
| CHA_2_DS_2_-VA points | |  |  |
| 0 | 0.4 (0.3-0.7) | 0.4 (0.2-0.6) | 0.5 (0.3-0.8) |
| 1 | 0.9 (0.7-1.2) | 0.9 (0.7-1.2) | 1.4 (1.1-1.8) |
| 2 | 2.8 (2.4-3.3) | 2.5 (2.1-3.0) | 2.6 (2.1-3.2) |
| 3 | 5.3 (4.7-5.9) | 5.4 (4.7-6.1) | 5.0 (4.3-5.7) |
| 4 | 8.6 (7.7-9.6) | 8.4 (7.5-9.4) | 7.8 (6.9-8.9) |
| 5 | 17.8 (16.0-19.9) | 13.8 (12.3-15.4) | 11.6 (10.2-13.1) |
| 6 | 23.7 (20.3-27.4) | 17.5 (15.1-20.3) | 18.2 (15.8-20.9) |
| 7 | 22.4 (17.0-29.0) | 16.5 (12.8-20.9) | 17.0 (13.2-21.4) |
| 8 | 24.9 (13.2-42.5) | 22.2 (13.1-35.1) | 18.6 (11.5-28.4) |
| Abbreviations: CHA_2_DS_2_-VA(Sc), congestive heart failure (1 point), hypertension (1 point), age ≥75 years (2 points), diabetes (1 point), history of stroke or transient ischemic attack (2 points), vascular disease (1 point), age 65-74 years (1 point), sex category (female) (1 point). 95% confidence intervals in parenthesis. | | | |

**Supplementary Figure 1.** Results of the sensitivity analyses with the cohort divided into four-year intervals. Temporal trends of continuous net reclassification index (panel A), category-based net reclassification index (panel B), integrated discrimination improvement (panel C) and c-statistics of the proportional hazards models (panel D).

In panels A-C, the red horizontal dashed line at zero level represents the CHA_2_DS_2_-VASc score as the reference. Values below this line are in favor of the CHA_2_DS_2_-VASc score, while values above favor the CHA_2_DS_2_-VA score. Error bars represent 95% confidence intervals. 1% and 2% thresholds were used to separate low-, moderate-, and high- risk categories in the category-based net reclassification index. p-values for differences in the proportional hazards models 0·0010, 0·084 and <0·0001 for calendar year periods 2007-2010, 2011-2014 and 2015-2018, respectively.

**Supplementary Figure 2.** Results of the sensitivity analyses with a two-year follow-up. Temporal trends of continuous net reclassification index (panel A), category-based net reclassification index (panel B), integrated discrimination improvement (panel C) and c-statistics of the proportional hazards models (panel D).

In panels A-C, the red horizontal dashed line at zero level represents the CHA_2_DS_2_-VASc score as the reference. Values below this line are in favor of the CHA_2_DS_2_-VASc score, while values above favor the CHA_2_DS_2_-VA score. Error bars represent 95% confidence intervals. 1% and 2% thresholds were used to separate low-, moderate-, and high- risk categories in the category-based net reclassification index. p-values for differences in the proportional hazards models 0·15, 0·0087, 0·42, 0·070, 0.0053 and 0·0028 for calendar year periods 2007-2008, 2009-2010, 2011-2012, 2013-2014, 2015-2016 and 2017-2018, respectively.
